# A multicenter retrospective analysis: Factors influencing hepatic adverse events induced by immunotherapy in advanced liver cancer

**DOI:** 10.1002/cnr2.1918

**Published:** 2023-12-11

**Authors:** Rui Zou, Yunhe Hao, Yiyao Wang, Feihu Yan, Xu Peng, Zepeng Huang, Gang Chen

**Affiliations:** ^1^ Department of Hepatobiliary and Pancreatic Surgery Hainan Cancer Hospital Haikou China; ^2^ Department of Oncology Chengmei Hospital Haikou Hainan Province China

**Keywords:** immune checkpoint inhibitor, immune‐related adverse events, liver cancer immune therapy, liver toxicity

## Abstract

**Objectives:**

To analyze the clinical characteristics and influencing factors of hepatotoxicity in patients with advanced hepatocellular carcinoma (HCC) treated with programmed cell death protein‐1 (PD‐1) inhibitors, and to provide a theoretical basis for the treatment of immune‐related hepatotoxicity in patients with advanced HCC.

**Methods:**

Retrospective analysis of clinical data of patients with advanced HCC from February 2021 to February 2023, in order to summarize and statistically analyze the influencing factors of immune‐related liver adverse reactions.

**Results:**

A total of 135 patients met the inclusion criteria, among whom 46 patients experienced varying degrees of immune‐related liver adverse reactions, with an incidence rate of 34.1% (46/135). The time range of immune‐related liver adverse reactions was 3–26 weeks, with a median time of 4 weeks. The age range of immune‐related liver adverse reactions was 34–73 years, with a median age of 62 years. Statistical analysis of the influencing factors and liver adverse reactions showed that age, total bilirubin level, and Child–Pugh (C–P) grading were influencing factors for the occurrence of liver adverse reactions (*p* < .05), and among these three influencing factors, the proportion of males with ≥2 influencing factors was higher than that of females; liver function C–P B was an independent influencing factor for liver adverse reactions (*p* < .05).

**Conclusion:**

For male patients over 60 years old, with bilirubin levels ≥51 μmol/L and liver function C–P B, close observation of the occurrence of immune‐related adverse reactions during treatment is recommended.

## 1 INTRODUCTION

Immunotherapy, particularly immune‐based combination therapy or single‐agent immune checkpoint inhibitors, has demonstrated potential and unprecedented effects in adjuvant therapy for advanced liver cancer.^1^, ^2^ Studies have shown^3^ that the application of immune therapy alone or in combination with other drugs significantly increases the opportunity for achieving complete remission. However, the occurrence of immune‐related adverse events is a critical factor that affects both the efficacy of immune therapy and patient prognosis. Therefore, this study aims to provide a theoretical basis for the prevention and treatment of immune‐related liver toxicity by retrospectively analyzing the treatment process of advanced liver cancer in our hospital and statistically analyzing the influencing factors of immune‐related liver adverse events.

## 2 MATERIALS AND METHODS

### 2.1 Clinical data

A retrospective analysis was conducted on 135 patients with advanced hepatocellular carcinoma (HCC) who were treated with PD‐1 inhibitors from September 2018 to September 2022. Clinical data including gender, age, smoking and alcohol consumption, hepatitis B status, hepatitis B DNA, alanine transaminase (ALT), aspartate aminotransferase (AST), hepatitis C status, alkaline phosphatase (ALP), total bilirubin, portal vein tumor thrombosis, alpha‐fetoprotein (AFP), abnormal coagulation function, Child–Pugh grading, treatment methods (monotherapy or combination targeted therapy), and adverse reactions were collected.

### 2.2 Inclusion and exclusion criteria

All patients were diagnosed with primary HCC. Patients were staged based on Child–Pugh grading, physical performance status, tumor size, vascular and extrahepatic metastasis, with staging between CNLC stage IIb and stage IIIb. Before treatment, liver function and bilirubin levels were normal. First‐line treatment consisted of PD‐1 inhibitors (pembrolizumab 2 mg/kg every 3 weeks; nivolumab 3 mg/kg every 2 weeks; camrelizumab 200 mg every 2 weeks; sintilimab 200 mg every 3 weeks) for at least four cycles, with some patients also receiving lenvatinib. Informed consent was obtained from all patients before treatment. Patients with autoimmune diseases, diabetes, hyperthyroidism, or cardiorespiratory dysfunction were excluded.

### 2.3 Hepatotoxicity assessment

According to the “Chinese Society of Clinical Oncology (CSCO) Guidelines for the Management of Immune Checkpoint Inhibitor‐Related Hepatotoxicity, 2021,”^4^ immune‐related hepatotoxicity occurring during treatment was graded and recorded. Grade 1 (G1): AST or ALT <3 times the upper limit of normal (ULN) and total bilirubin <1.5 times ULN; Grade 2 (G2): AST or ALT 3–5 times ULN and total bilirubin 1.5–3 times ULN; Grade 3 (G3): AST or ALT 5–20 times ULN and total bilirubin 3–10 times ULN; Grade 4 (G4): AST or ALT >20 times ULN and total bilirubin >10 times ULN.

### 2.4 Principles of hepatotoxicity treatment

G1: Liver function changes were monitored weekly, and immunotherapy was continued. G2: Immunotherapy was temporarily discontinued, and prednisone was orally administered at a dose of 0.5–1 mg/kg/day. After liver function improvement, prednisone was gradually tapered with liver function monitoring every 3 days for a total treatment duration of 4 weeks. When prednisone was reduced to 10 mg/day and hepatotoxicity decreased by ≤1 grade, immunotherapy could be resumed. G3: Treatment method was similar to G2, but monitoring frequency was increased to once every 1–2 days. If liver function did not improve, additional medications such as macitentan (500–1000 mg, twice daily) and tacrolimus were added. G4: Permanent discontinuation of immunotherapy and intravenous administration of methylprednisolone at a dose of 1–2 mg/kg. After hepatotoxicity decreased to G2, oral administration was initiated. The total treatment duration was at least 4 weeks. If there was no improvement after 3 days, additional medications such as macitentan (500–1000 mg, twice daily) and tacrolimus were added. It is important to note that during the management of adverse reactions, proton pump inhibitors should be administered routinely to protect gastric mucosa when the expected duration of hormone or immunosuppressant treatment exceeds 3 weeks (or prednisone >30 mg/kg/day).

### 2.5 Statistical methods

Statistical analysis was performed using SPSS version 26.0 software. The chi‐square test was used for categorical data, *t*‐tests for normally distributed continuous data, analysis of variance for between‐group comparisons, and logistic regression analysis for multivariable analysis. A *p*‐value <.05 was considered statistically significant.

## 3 RESULTS

### 3.1 Incidence of hepatic adverse reactions

Among the 135 patients with HCC who received immunotherapy, 46 cases experienced immunotherapy‐related hepatic toxicity, resulting in an incidence rate of 34.1% (46/135). Among these cases, there were 33 males and 13 females, with ages ranging from 34 to 73 years and a median age of 62 years. The onset of hepatic adverse reactions occurred within 3–26 weeks, with a median time of 25 days. The grading of hepatic adverse reactions in the 46 cases was assessed according to the immunotherapy guidelines for HCC patients (Table 1).

**TABLE 1:** Grading of hepatic adverse reactions in hepatocellular carcinoma patients (*n* = 46).

Grading	Levels of AST, ALT, and total bilirubin	Liver adverse events (case)
G1	AST or ALT <3 times ULN	11
	Total bilirubin <1.5 times ULN	
G2	AST or ALT 3–5 times ULN	27
	Total bilirubin 1.5–3 times ULN	
G3	AST or ALT 5–20 times ULN	6
	Total bilirubin 3–10 times ULN	
G4	AST or ALT >20 times ULN	2
	Total bilirubin >10 times ULN	

### 3.2 Factors related to hepatic adverse reactions

#### 3.2.1 Relationship between total bilirubin levels Child–Pugh classification, and hepatotoxicity grading

**FIGURE 1:**
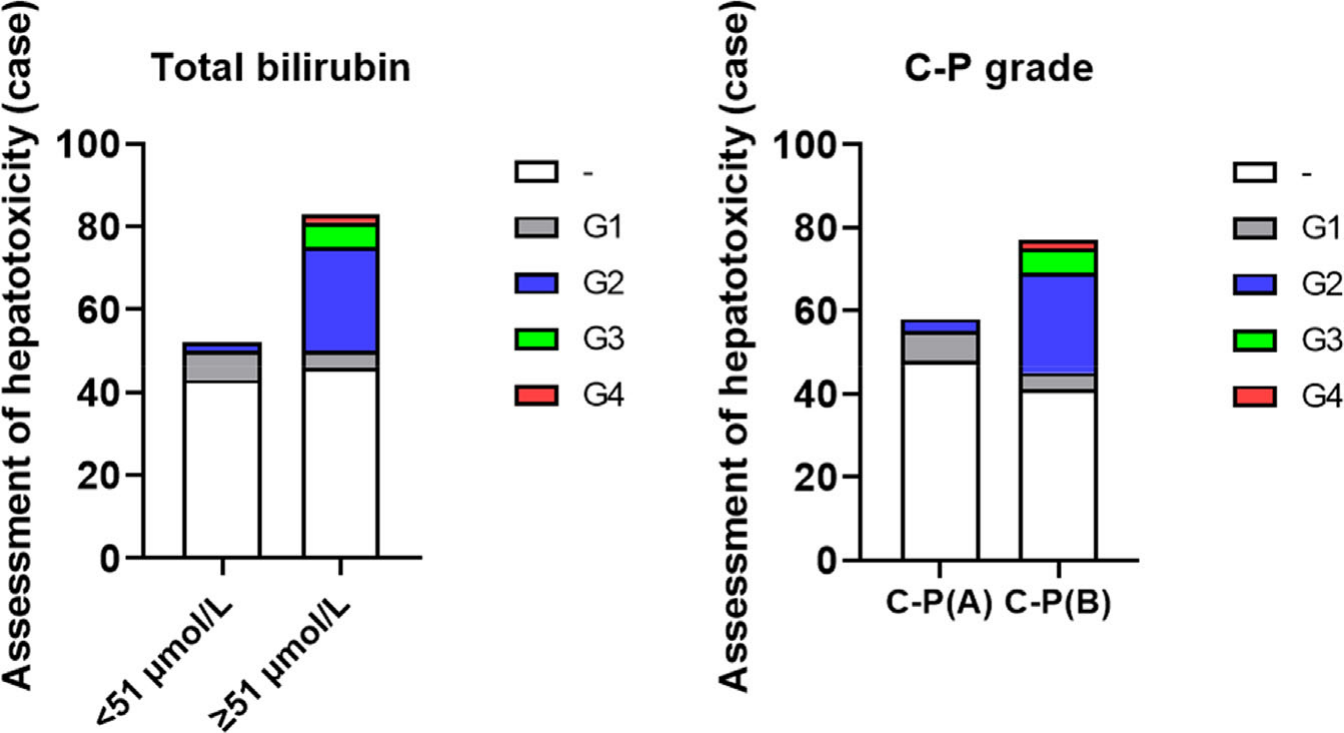
Total bilirubin levels and Child–Pugh score affect the occurrence of liver adverse events.

The number of patients corresponding to hepatotoxicity grading based on total bilirubin levels and Child–Pugh classification can be seen in Figure 1. Univariate analysis showed that age, total bilirubin levels, and Child–Pugh classification were the main factors influencing the occurrence of hepatic adverse reactions in HCC patients receiving immune checkpoint inhibitor treatment (*p* < .05, Table 2). Logistic regression multivariate analysis indicated that liver function classified as Child–Pugh grade B was an independent factor influencing the occurrence of hepatic adverse reactions in patients (*p* < .05, Table 3). The average age as a factor affecting the occurrence of hepatic adverse reactions was 58.15 years with a median age of 58 years, while the average age in the hepatic adverse reaction group was 61.52 years with a median age of 63 years.

**TABLE 2:** Univariate analysis of factors influencing adverse liver reactions (*n* = 135).

Factors influenced		Patients (cases)	Adverse liver reactions (cases)	*p*‐Value
Gender	Male	84	33	0.1023
	Female	51	13	
Age	<60	57	10	0.0004
	≥60	78	36	
Smoking	Yes	17	4	0.3302
	No	118	42	
Alcohol abuse	Yes	12	3	0.4908
	No	123	43	
HBV	Yes	99	32	0.4803
	No	36	14	
HBV‐DNA	<1000 cps/mL	77	27	0.626
	≥1000 cps/mL	58	19	
HCV	Yes	2	2	1.024
	No	133	44	
ALT	<42 μ/L	122	42	0.827
	≥42 μ/L	13	4	
AST	<44 μ/L	120	40	0.726
	≥44 μ/L	15	6	
ALP	<32 μ/L	60	17	0.211
	≥32 μ/L	75	29	
Total bilirubin	<51 μmol/L	53	9	0.0006
	≥51 μmol/L	82	37	
Portal vein thrombosis	Yes	34	11	0.8083
	No	101	35	
AFP	<25 μg/L	47	15	0.7015
	≥25 μg/L	88	31	
Abnormal prothrombin	<20 μg/L	64	21	0.7711
	≥20 μg/L	71	25	
Child–Pugh	A	42	9	0.0375
	B	93	37	
Treatment	Monotherapy	16	5	0.8014
	Combination therapy	119	41	

**TABLE 3:** Multivariable logistic regression analysis of hepatic adverse reactions.

Factors influenced	*β*	Wald	OR	95% CI		*p*
				Lower limit	Upper limit	
C–P	1.218	6.466	3.381	1.322	8.645	0.011
Age	0.039	3.411	1.040	0.998	1.084	0.065
Total bilirubin	0.010	0.841	1.010	0.989	1.032	0.359

#### 3.2.2 Relationship between age, total bilirubin levels, C–P B level, and gender

**FIGURE 2:**
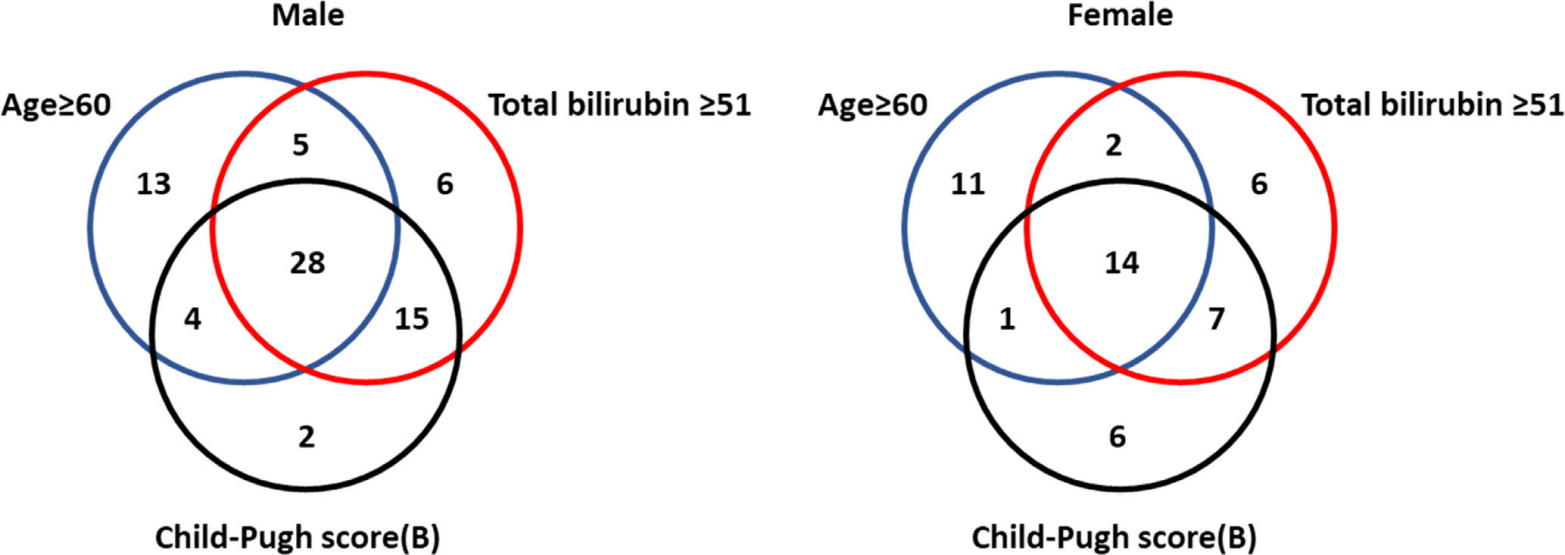
Proportion analysis of age, total bilirubin levels, and C–P B level in male and female patients.

In order to examine the relationship between age, total bilirubin levels, C–P B level, and gender, we compared the proportions of individuals with two or more influencing factors in different genders using Venn diagram analysis. Among these influencing factors, the proportion of male patients meeting the criteria was 71.2% (52 out of 73), while the proportion of female patients meeting the criteria was 51.1% (24 out of 47; Figure 2).

### 3.3 Clinical characteristics and treatment response of different liver adverse reactions

In 135 patients, 46 developed liver adverse reactions, among which 8 cases (17.39%, 8/46) experienced Grade 3 or higher liver adverse reactions. All patients with liver adverse reactions exhibited elevated levels of AST, ALT, and total bilirubin. In the eight severe liver adverse reaction cases (Table 4), total bilirubin levels were significantly higher than twice the normal value (≥51 μmol/L). Among the eight cases of severe liver adverse reactions, six were Grade 3, and two were Grade 4, all of which were treated with methylprednisolone (intravenous injection, 1–2 mg/kg/day). Among them, three patients showed significant improvement of adverse reactions after 1 week of methylprednisolone treatment, with simultaneous decrease in ALT, AST, and total bilirubin levels.

**TABLE 4:** Clinical characteristics of eight patients with severe liver adverse reactions.

Example	Gender	Age (years)	Smoking	Alcohol abuse	HBV	HBV‐DNA (103 cps/mL)	Total bilirubin (μmoL/mL)	C–P	Adverse reaction	Grade efficacy
1	Male	37	Yes	Yes	(−)	‐	92	B	G3	Gradual improvement observed after 1 week of treatment
2	Male	53	Yes	No	(+)	1.54	64	B	G3	Gradual improvement observed after 2 week of treatment
3	Male	60	No	No	(+)	182	115	B	G4	Gradual improvement observed after 7 weeks of treatment, leading to discontinuation of immunotherapy
4	Male	64	No	No	(−)	‐	74	B	G3	Gradual improvement observed after 1 week of treatment
5	Male	65	No	No	(+)	62.2	107	B	G3	Gradual improvement observed after 3 weeks of treatment, but the patient refused further treatment
6	Male	69	No	No	(−)	‐	57	B	G3	Gradual improvement observed after 1 week of treatment
7	Male	70	No	No	(−)	‐	75	B	G4	No improvement observed after 7 weeks, switched to treatment with tacrolimus for 4 weeks leading to improvement, resulting in discontinuation of immunotherapy
8	Female	61	No	No	(+)	1680	77	B	G3	Gradual improvement observed after 3 week of treatment

## 4 DISCUSSION

The method of treating tumors by blocking the interaction between PD‐1 and PD‐L1 was first published in 2001.^5^ In recent years, tumor immunotherapy has become an important method for treating malignant tumors. Immune checkpoint inhibitors activate the immune response by relieving the inhibitory effects of tumor cells on T cells, with the aim of killing tumor cells. However, during tumor immunotherapy, overactivation of T cells can cause autoimmune diseases, leading to a series of treatment‐related adverse reactions.^6^, ^7^ Liver adverse events are one of the adverse events associated with immunotherapy,^8^, ^9^ and this is more likely to occur in patients with liver cancer.^10^ Research has indicated that^11^ Tregs, characterized by the expression of the Foxp3 transcription factor, are the most abundant inhibitory cells in the tumor microenvironment and are associated with tumor progression and metastasis. Tregs express checkpoint molecules, which are direct targets of immune checkpoint inhibitors in immunotherapy, but there is also a risk of immune‐related adverse events due to the disruption of immune balance.

According to studies, severe liver‐related adverse events have been observed in 4% of patients receiving immune checkpoint inhibitor therapy.^12^ In this study, the occurrence rate of liver adverse events in patients receiving PD‐1 immune checkpoint inhibitor therapy was 34.1% (46/135), with a severe (G3, G4) liver toxicity rate of 5.9% (8/135).^13^ Besides liver tumors, a history of autoimmune diseases and non‐alcoholic fatty liver disease are also risk factors for immune therapy‐induced liver adverse events.^14^, ^15^ Thus, liver inflammation and the occurrence of adverse reactions are closely related. The univariate analysis in this study showed that age, total bilirubin, and C–P B grade were influencing factors for liver toxicity occurrence. Immune checkpoint inhibitors enhance the cytotoxic effect of T cells against tumor cells.^16^ However, the enhanced cell‐mediated immunity also attacks self‐tissues, leading to the occurrence of autoimmune diseases accompanied by enhanced humoral immunity and the production of corresponding inflammatory factors, further exacerbating the development of diseases.^17^, ^18^, ^19^ Age, as a major risk factor for human health, increases the risk of autoimmune diseases due to immune system disruption and aging‐related changes in secretory phenotypes that lead to a chronic inflammatory state.^20^, ^21^, ^22^, ^23^ In this study, we found that age is a factor contributing to immune suppression therapy‐induced liver adverse events, mainly because age is closely related to immune status. Moreover, high levels of total bilirubin (≥51 μmol/L) and Child–Pugh B score can reflect changes in the degree of body inflammation. Therefore, based on the data from this study, age, total bilirubin, and Child–Pugh score are closely related to the degree of self‐immunity and immune suppression therapy‐induced liver adverse events.

The multivariate analysis in this study showed that liver function C–P B grade is an independent factor influencing the occurrence of liver adverse events in patients (*p* < .05), which has not been reported domestically or internationally. This study is a retrospective study based on clinical practice and has limitations such as a small sample size and multiple confounding factors. Therefore, well‐designed multicenter prospective studies are still needed to further confirm the findings.

Previous studies have suggested that gender is a factor influencing liver adverse events, with female patients being more prone to severe liver adverse reactions.^24^ However, in this study, male patients were more likely to experience liver adverse reactions than female patients, although the difference was not statistically significant in univariate and multivariate analyses (*p* = .1023). Our proportion analysis of the three influencing factors, age, bilirubin, and Child–Pugh B grade, in male and female patients showed that the proportion of male patients (71.2%, 52/73) was significantly higher than that of female patients (51.1%, 24/47), which may explain the higher likelihood of liver adverse events in male patients.

For patients who experience liver adverse events, the focus of treatment is to reduce inflammation and suppress immune responses. Liver adverse events are mainly caused by excessive activation of T cells, and the accompanying enhancement of humoral immunity can exacerbate adverse events through the production of inflammatory factors. Therefore, inhibiting the immune system, reducing inflammation, and improving excessive cell‐mediated immunity can alleviate liver adverse events.^25^, ^26^, ^27^ In this study, one patient with G4 liver toxicity showed symptom improvement after using tacrolimus.

In conclusion, the management and prevention of liver adverse events caused by immune therapy are crucial. We have preliminarily explored possible influencing factors and recommend that all clinical doctors pay attention to toxicity management and strictly adhere to indications. For elderly patients, patients with elevated bilirubin levels, and patients with C–P B grade, special attention should be given to the occurrence of immune‐related liver toxicity. However, due to the limitations of this single‐center retrospective study, larger‐scale prospective studies are recommended to explore ways to prevent liver adverse events. It is believed that this study will play a crucial role in the prevention of liver adverse events in immunotherapy for liver cancer in the next 5 years.

## AUTHOR CONTRIBUTIONS

**Rui Zou:** Methodology (equal); writing – review and editing (lead). **Yiyao Wang:** Data curation (lead); writing – original draft (lead). **Feihu Yan:** Data curation (equal); software (equal); writing – original draft (equal).

## FUNDING INFORMATION

Key R&D Projects in Hainan Province (ZDYF2022SHFZ118), Research Fund Project of Hainan Cancer Hospital (2023ZD05), Nanhai Xinxing Medical and Health Talent Platform Project.

## ETHICS STATEMENT

The Research Ethics Committee of Hainan Cancer Hospital has approved the plan (SEC‐2022‐016‐01).

## Data Availability

N/A.
